# Pathological and therapeutic roles of bioactive peptide trefoil factor 3 in diverse diseases: recent progress and perspective

**DOI:** 10.1038/s41419-022-04504-6

**Published:** 2022-01-17

**Authors:** Yiqi Yang, Ziyang Lin, Quanyou Lin, Weijian Bei, Jiao Guo

**Affiliations:** Key Laboratory of Glucolipid Metabolic Diseases of the Ministry of Education, Guangdong Metabolic Diseases Research Center of Integrated Chinese and Western Medicine, Guangdong Pharmaceutical University, Science and Technology Building, 280 Waihuan East Road, Guangzhou Higher Education Mega, Guangzhou, China

**Keywords:** Biochemistry, Cell biology

## Abstract

Trefoil factor 3 (TFF3) is the last small-molecule peptide found in the trefoil factor family, which is mainly secreted by intestinal goblet cells and exerts mucosal repair effect in the gastrointestinal tract. Emerging evidence indicated that the TFF3 expression profile and biological effects changed significantly in pathological states such as cancer, colitis, gastric ulcer, diabetes mellitus, non-alcoholic fatty liver disease, and nervous system disease. More importantly, mucosal protection would no longer be the only effect of TFF3, it gradually exhibits carcinogenic activity and potential regulatory effect of nervous and endocrine systems, but the inner mechanisms remain unclear. Understanding the molecular function of TFF3 in specific diseases might provide a new insight for the clinical development of novel therapeutic strategies. This review provides an up-to-date overview of the pathological effects of TFF3 in different disease and discusses the binding proteins, signaling pathways, and clinical application.

## Facts

- TFF3 is the driving factor of cancer, which is involved in the proliferation, invasion, resistance to apoptosis, and angiogenesis of cancer cells.
- TFF3 is a promising novel neuropeptide that regulates diverse brain functions.
- Hepatic TFF3 may serve as therapeutic targets for glycolipid metabolic diseases.
- TFF3 is widely distributed in human body, and is also present as a mixed ligand, regulating multiple signal pathways.

## Open questions

- Which upstream links contribute to dysregulation of TFF3 expression during cancer progression?
- What factors determine the carcinogenic effect of TFF3, and how is the balance between mucosal protection and carcinogenesis achieved?
- How does TFF3 participate in the innate immune protection of the host and can it regulate the proliferation and differentiation of immune cells?
- What factors hinder the clinical efficacy of TFF3 in colitis?

## Introduction

Trefoil factor peptides (TFFs) are small molecular peptides secreted by goblet cells and epithelial cells of various tissues in mammals. The trefoil factor family comprises three members, namely gastric peptide (TFF1 or pS2), spasmolytic polypeptide (TFF2 or SP), and intestinal trefoil factor (TFF3 or ITF). TFF3 contains 59 amino acids and a seventh free cysteine residue at position 57, which is crucial for dimer formation [1]. Although dimerization is not necessary for TFF function, the dimer is the more potent form than the monomer. For instance, TFF1 dimers promoted cell migration eight times more efficiently than the monomers [2]. Intracolonic injection of TFF3 dimers (but not monomers) ameliorates experimental colitis in rats [3]. The antiapoptotic effect of TFF3 depends on the intact dimerized peptide [4].

Recent studies have reported that TFF3 is present in not only the intestinal tract but also the brain [5], liver [6], kidney [7], pancreas [8], breast [9], lung [10], conjunctiva [11], spleen, and lymph nodes [12] (Table 1). It participates in the pathological process of various diseases such as type 2 diabetes mellitus (T2DM), non-alcoholic fatty liver disease (NAFLD), neurodegeneration, gastric ulcer, colitis, and cancer. However, the biological functions and specific mechanisms of TFF3 expressed in these tissues remain unclear. Despite consistent reports regarding the highly promising mucosal healing capacity of TFF3 [13], till date, drugs developed using TFF3 have not made significant progress in clinical practice. For a long time, several attempts to isolate TFF-binding proteins with typical receptor characteristics have failed [14], leading to considerable gaps in our understanding of TFF3 at the molecular level. These gaps hinder its application and development in clinical medicine. In this review, we present and discuss the biological effects, binding proteins, proposed mechanisms, and clinical applications of TFF3 in currently researched diseases.

**Table. 1:** TFF3 distribution in samples from healthy humans.

Category	Expression site	Detection method	Reference
Digestive system	Intestine	PCR, RT-PCR, WB, IHC	[123, 124]
	Salivary gland	PCR, RT-PCR	[123]
	Liver	PCR, IHC	[6, 123]
	Stomach	PCR, RT-PCR, WB, IHC	[51, 123, 124]
	Esophagus, gall bladder, and bile ducts	IHC	[123]
	Vater’s ampulla	RT-PCR, WB	[123, 124]
	Submandibular glands	RT-PCR, WB, IF	[125]
	Sublingual glands	IHC, RT-PCR	[123, 125]
Female reproductive system	Mammary gland	PCR, RT-PCR	[123]
	Uterus	PCR, IHC	[123]
	Endocervix	RT-PCR, WB, IF	[126]
	Endometrium	RT-PCR	[126]
	Vagina	IHC	[123]
	Breast	ISH, IHC	[9]
	Human milk	ELISA	[127]
Urinary system	Prostate	PCR, RT-PCR	[123]
	Urinary tract	RT-PCR, IF	[7]
	Renal medulla	RT-PCR, IF, WB	[7]
	Urethra	RT-PCR, IF	[7]
	Renal pelvis, bladder trigone, and ureter	IF	[7]
Respiratory system	Trachea	PCR, RT-PCR, IHC, WB	[10, 121, 123]
	Lung	PCR, RT-PCR	[10, 121, 123]
Central nervous system	Hypothalamus	IHC, RT-PCR	[5, 35, 36]
	Neurohypophysis	WB, PT-PCR	[5, 35, 36]
	Cerebrospinal fluid	WB	[35]
	Cerebral cortex, hippocampus, amygdala, basal ganglia, thalamus, cerebellum, midbrain, brain stem, white matter, choroid plexus	WB	[5]
Immune system	Spleen	PCR	[123]
Endocrine System	Pancreas	PCR, RT-PCR, IHC, ELISA	[8, 123]
	Thyroid gland	IHC	[123]
Ocular system	Conjunctiva	RT-PCR, IF, IHC	[11, 128]
	Efferent tear duct	RT-PCR, IHC	[129]
	Nasolacrimal duct	WB, IHC	[124, 129]
	Lacrimal sac	WB, IHC	[129]
	Corneae	RT-PCR	[130]

*WB* western blot, *IHC* immunohistochemistry, IF immunofluorescence, *ISH* in situ hybridization, RT-PCR reverse transcription-polymerase chain reaction, ELISA, enzyme linked immunosorbent assay.

## Disease phenotypes in TFF3 deficiency mice

Colitis is the first and most concerning disease to be studied using TFF3^−/−^ mice. Since TFF3 plays an important role in the protection of the intestinal barrier, its deficiency not only renders the body particularly sensitive to glucan sodium sulfate, chemotherapy, and radiation-induced mucosal damage, but also induces markedly poor epithelial regeneration, leading to extensive death of mice from colitis [15, 16]. Similarly, epithelial regeneration defects were observed in corneal injury models of TFF3^−/−^ mice, and the completed wound healing extended to 462 h after corneal injury, which almost 5 times of wild-type mice (98 h); while exogenous TFF3 administration can accelerate corneal wound healing in a dose-dependent manner in vivo [17]. Interestingly, disruption of the TFF3 gene does not affect the normal morphological development of the cornea [17]. It has been suggested that TFF3 is required for corneal wound healing but not for physiological maintenance of the corneal epithelium. This is of great significance for the treatment of infectious keratitis induced by delayed epithelial healing after excimer laser surgery.

Metabolic diseases and brain diseases are new directions in TFF3^−/−^ mice research in recent years. Compared with wild-type mice, TFF3^−/−^ mice exhibited not only a lower body weight but also significant dysregulation of 21 miRNAs [18]. Most of these miRNAs were related to the metabolic pathway “glycolysis/gluconeogenesis” [18]. Further research indicated that adenovirus-induced TFF3 knockdown leads to a pronounced fatty-liver phenotype [19]. Conversely, intraperitoneal injection of recombinant TFF3 protein or tail-vein injection of adenovirus-mediated TFF3 overexpression ameliorated several indicators associated with NAFLD in diabetic and obese mice. Furthermore, TFF3 deficiency may also affect vascular function and stroke outcome. In a model of brain injury induced by transient occlusion of the middle cerebral artery, high-salt diet resulted in significant reduction in endothelium-dependent vasodilation of carotid arteries in TFF3^−/−^ mice, and their cerebral infarction area was larger than that in wild-type mice [20]. Furthermore, compared with wild-type mice, experimental cerebral ischemia/reperfusion TFF3^−/−^ mice exhibited higher caspase 3 activity and cell death, larger cerebral infarction area, and more severe forelimb motor defects [21]. Intravenous administration of recombinant TFF3 can reverse brain damage and TFF3-deficiency-induced changes in forelimb motor function [21]. The above results suggested that TFF3^−/−^ mice could be used as a potential new model for glycolipid metabolism and cerebrovascular function research.

In summary, phenotypic changes caused by TFF3 deficiency include intestinal epithelial and corneal regeneration defects, accelerated presbyopia and hearing impairment, weight loss, abnormal lipid metabolism, and impaired vascular function (Table 2). TFF3^−/−^ mice may be a useful model to study the pathogenic process of inflammatory enteritis, stroke, NAFLD and keratitis, and it is of great significance for disease treatment and drug development targeting TFF3.

**Table. 2:** Disease phenotypes resulting from TFF3 deficiency.

Research direction	Modeling method	Disease	Phenotypic changes	Reference
Gastrointestinal research	Oral dextran sulfate sodium	Colitis	Poor epithelial regeneration leading to widespread death from colitis.	[15]
	Radiation and chemotherapy	Intestinal mucositis	It is very sensitive to chemotherapy- and radiotherapy-induced mucositis.	[16]
	Oral dextran sulfate sodium	Colitis	Leads to complete suppression of TLR2-mediated anti-apoptosis in acute mucosal inflammation	[67]
			Colonic apoptosis increased, but expression of receptor-related or stress-related cell death regulators did not change significantly.	[131]
	Oral Toxoplasma gondii	Ileitis	IFN-γ, IL-12, IL-1β, and TNF-α gene expression decreased, and ileal CD4^+^ lymphocyte count reduced.	[132]
			It induced decrease in TFF1 and TFF2 levels in the stomach. Trefoil peptides may individually regulate transcription of the entire family.	[133]
Metabolism research			Body weight was significantly lower than that of wild-type mice. Metabolic-pathway-related genes were significantly dysregulated.	[18]
			Accumulation of fatty acids in the liver was affected, and metabolism-related gene expression was reduced.	[33]
	Injection of tunicamycin	Acute endoplasmic reticulum stress	Decreased ability for pro-inflammatory cascade initiation.	[34]
Nervous system research	High-salt diet combined with transient occlusion of middle cerebral artery	Stroke	TFF3 depletion impaired vascular function and worsened stroke outcomes.	[20]
	Ischemia/reperfusion	Brain Injury	Cell death, cerebral infarction, and forelimb motor deficits were more severe.	[21]
	Alkali and laser	Corneal Injury	Re-epithelialization of corneal wounds is significantly prolonged.	[17]
			Exhibited accelerated presbycusis and more pronounced high-frequency hearing loss.	[39]
	Loudspeaker	Hearing impaired	Presbycusis-related gene expression was significantly downregulated by TFF3 deletion.	[40]

*TLR* toll-like receptor, *IFN-γ* interferon-γ, *IL* interleukin, *TNF-α* tumor necrosis factor-α.

## Pathological effects of TFF3 in disease

### TFF3 in T2DM

**Fig. 1:**
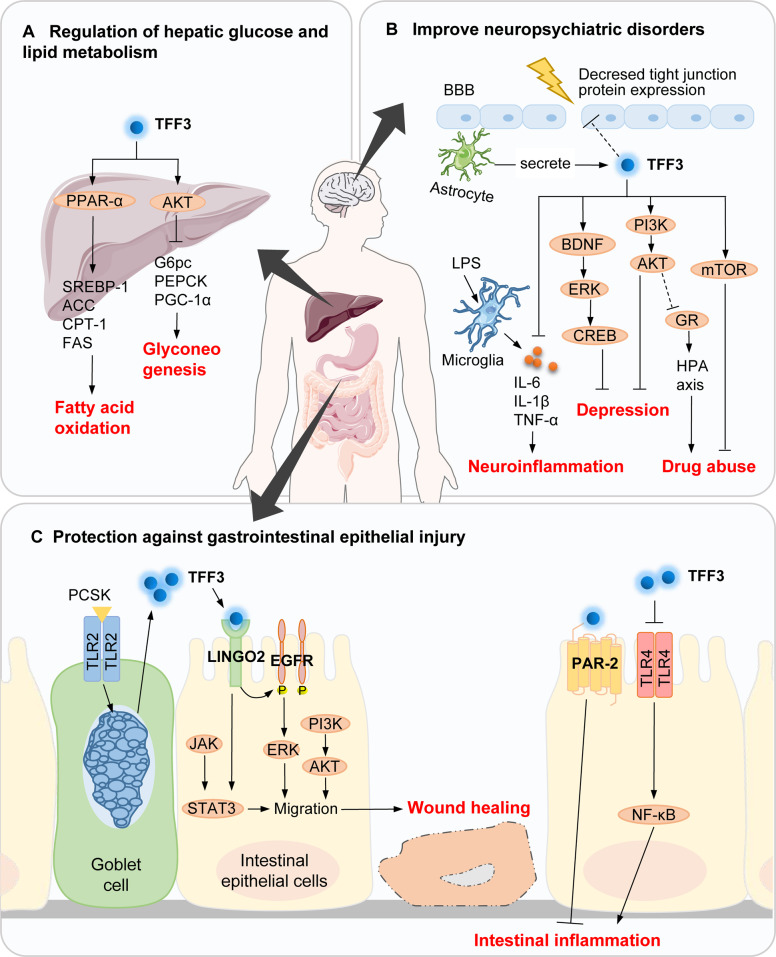
Mechanisms of TFF3 in metabolic diseases, nervous system diseases, and gastrointestinal disorders. **A** In the liver, TFF3 regulates fatty-acid oxidation and gluconeogenesis-related proteins through the PPAR-α and AKT pathways, respectively, to ameliorate T2DM and NAFLD. **B** TFF3 can regulate occludin, claudin-1, and ZO-1. These major tight-junction proteins of the blood–brain barrier may be downregulated because of age, inflammation, and stress, triggering nervous-system diseases. TFF3 additionally exerts anti-neuroinflammatory, antidepressant, and anti-drug-addictive properties; the underlying mechanisms are related to the BNDF/ERK/CREB, PI3K/AKT, mTOR, and GR signaling pathways, as well as the suppression of inflammatory secretion by microglia. Systemic TFF3 administration or induction of TFF3 secretion from astrocytes could be potential treatments. **C** TLR2 ligands, such as synthetic Pam3CysSK4, stimulates TFF3 secretion from goblet cells, and TFF3 can interact with LINGO2 to enhance EGFR signaling and subsequently promote epithelial cell migration to achieve wound healing. Furthermore, TFF3 suppressed intestinal inflammation via PAR-2 and TLR4/NF-κB signaling. Established functions are denoted by solid arrows, whereas unidentified pathways are indicated by question marks. PPAR-α, peroxisome proliferator-activated receptor alpha; SREBP-1, sterol regulatory element binding protein 1; ACC, acetyl-CoA carboxylase; CPT-1, carnitine palmitoyltransferase 1; FAS, fatty acid synthase; G6PC, glucose-6 phosphatase catalytic subunit; PEPCK, phosphoenolpyruvate carboxykinase; PGC-1α, peroxisome proliferator-activated receptor-γ coactivator-1α; BBB, blood–brain barrier; LPS, lipopolysaccharide; IL, Interleukins, TNF-α, tumor necrosis factor; BDNF, brain-derived neurotrophic factor; ERK, extracellular signal-related kinase; CREB, cyclic adenosine monophosphate response element binding protein; PI3K, phosphatidylinositol 3-kinase; GR, glucocorticoid receptor; HPA axis, hypothalamic pituitary adrenal axis; mTOR, mammalian target of rapamycin; PCSK, Pam3CysSK4; TLR, toll-like receptors; LINGO2, leucine-rich repeat receptor and nogo interacting protein 2; EGFR, epidermal growth factor receptor; STAT, signal transducer and activator of transcription, PAR, protease-activated receptor; NF-κB: nuclear factor κB.

T2DM is a chronic disease that results from insulin resistance. It can lead to various complications, such as chronic kidney disease (CKD). Clinical studies have shown that the level of serum TFF3 increased significantly in patients with T2DM and its complications [22–25]. This change was more obvious in patients with CKD. The serum TFF3 can be used to predict the decline of glomerular filtration rate and occurrence of CKD. It is a valuable diagnostic marker for early CKD in dysglycemic populations [22]. Interestingly, glucose and insulin can dose-dependently enhance the secretion of TFF3 by intestinal epithelial cells [26]. Additionally, short-chain fatty acids can dose-dependently inhibit the synthesis and release of TFF3, which may be mediated by transcription [27]. Therefore, the increase in blood glucose and insulin and decrease in short-chain fatty acids in patients with T2DM may potentially lead to the elevation of serum TFF3. Adenoviral-vector-mediated TFF3 overexpression or daily injection of recombinant TFF3 protein in vivo can improve glucose tolerance and insulin sensitivity and reduce blood glucose levels in diabetic or obese mice [28, 29], possibly through a reduction in hepatic glucose output by inhibiting gluconeogenic genes expression via activation of the AKT signaling pathway [28, 29] (Fig. 1A). These studies suggested that TFF3 is involved in hepatic glucose homeostasis, which provided a promising new lead for developing therapies against the metabolic disorders associated with T2DM.

### TFF3 in NAFLD

NAFLD is the hepatic manifestation of metabolic syndrome, with a prevalence as high as 29.2% in China [30]. It is a metabolic disease involving fatty degeneration and lipid accumulation in liver cells induced by insulin resistance. Microarray analysis showed that TFF3 was one of the most affected liver genes in rodent models of early fatty liver and diabetes [28, 31]. TFF3 gene expression is significantly downregulated in the livers of db/db, ob/ob, and high-fat diet-induced obese mice and changes most dramatically (600-fold reduction) in the early diabetic stage of a multigene diabetic model called Tally Ho mice [32]. Thus, TFF3 is increasingly recognized as a novel participant in NAFLD.

TFF3 was detected in both cell culture medium and cell extracts after adenovirus-TFF3-infected primary mouse hepatocytes indicating that it is efficiently secreted from primary hepatocytes [28]. In contrast, TFF3 deficiency impairs fatty-acid distribution and accumulation in mouse liver, showing reduced expression of metabolism-related genes and increased number of small lipid vesicles [33, 34], raising the possibility that TFF3 affects hepatic lipid-droplet formation. Conversely, intraperitoneal injection of recombinant TFF3 protein or tail-vein injection of adenovirus-mediated TFF3 overexpression ameliorated several indicators associated with NAFLD in diabetic (db/db), obese (ob/ob), and diet-induced obese mice or rats, possibly by mediating hepatic fatty-acid oxidation via increasing peroxisome proliferator-activated receptor-α levels [19] (Fig. 1A). Thus, hepatic TFF3 may serve as a potential therapeutic target for NAFLD.

### TFF3 in nervous system disease

Neuropeptides, the most common signaling molecules in nervous system disease, regulate a wide range of brain functions. Recent studies have shown that TFF3 protein is a novel neuropeptide expressed in various brain regions in humans, including the hypothalamus, pituitary gland, hippocampus, temporal cortex, and cerebellum [5, 35, 36]. Clinical findings have shown that TFF3 is the strongest predictor of neurodegeneration across the spectrum of cerebral amyloidosis, with significant reductions in TFF3 levels observed in patients with vascular parkinsonism dementia, Parkinson’s disease dementia, and Alzheimer’s disease [37, 38]. Similarly, TFF3^−/−^ mice has been shown to induce hearing impairment, accelerated presbycusis, and worsened stroke outcomes [20, 39, 40]. These observations imply that TFF3 plays non-negligible roles in both physiological and pathological processes.

Indirect evidence proved that after systemic administration, TFF3 can cross the blood–brain barrier, enter the brain after 30 min of administration [41], and produce sustained long-term memory, anxiolytic, and antidepressant effects [41–44]. Injection of specific inhibitors of phosphoinositide 3-kinase (PI3K), extracellular signal-regulated kinase (ERK), or brain-derived neurotrophic factor (BDNF) blocked the antidepressant-like effects produced by TFF3 in rats [41, 44]. These results suggest that the antidepressant-like effect of TFF3 may be mediated by the PI3K/AKT signaling pathway in the basolateral amygdala, as well as the BDNF/ERK/cyclic-adenosine-monophosphate-response-element-binding protein signaling pathway in the hippocampal CA (Fig. 1B). In a passive-avoidance test, injection of different doses of TFF3 into the amygdala of rats appeared to affect anxiety bidirectionally in a dose-dependent manner. A low dose was anxiolytic, whereas a high dose produced an anxiogenic effect [42]. In addition to administering TFF3, inducing nerve cells to secrete TFF3 may act as a potential therapeutic approach. Astrocytes are a source of TFF3 in the central nervous system of rats; they can significantly reduce the secretion of TFF3 under stimulation by lipopolysaccharide [45]. In contrast, microglia cultured in the presence of TFF3 showed decreased secretion of pro-inflammatory cytokines such as inducible nitric oxide synthase, interleukin (IL)-6, IL-1β, and tumor necrosis factor-α (TNF-α) after lipopolysaccharide stimulation. Furthermore, TFF3 played a regulatory role in drug abuse. The underlying mechanisms involve the activation of mammalian target of rapamycin (mTOR) signaling in the nucleus accumbens shell [46] and down-regulation of the hypothalamic–pituitary–adrenal-axis activity through glucocorticoid receptors [47]. In summary, TFF3 can improve memory; regulate drug addiction; and produce anxiolytic, antidepressant, and anti-neuroinflammatory effects (Table 3).

**Table. 3:** Neuromodulatory activity of TFF3.

Modeling method	Animal type	Intervention methods and dosage	Effectiveness and mechanism	Reference
Novel-object recognition task and locomotor activity monitoring	Kunming mice	Recombinant human TFF3 (0.1 and 0.5 mg/kg, i.p.)	Improves learning and retention of new object recognition memory.	[43]
Naloxone induces morphine-dependent withdrawal syndrome	ICR mice	Recombinant human TFF3 (1.0 mg/kg, i.p.)	Attenuates withdrawal syndrome by down-regulating HPA axis activity and increasing neuronal activation in medial prefrontal cortex.	[47]
Cocaine-induced hyperlocomotion and conditioned place preference	SD rats	Recombinant human TFF3 (0.01 or 0.1 mg/kg, i.p.)	Enhances cocaine-induced hyperlocomotion and conditioned place preference via mTOR signaling pathway.	[46]
Passive-avoidance behavior test	Wistar rats	Recombinant human TFF3 monomer (2 × 6 and 2 × 60 pg, inject bilaterally into amygdala)	Bidirectionally affects anxiety in a dose-dependent manner. Low dose is anxiolytic, while high dose is anxiety-causing.	[42]
Forced swim test, tail suspension, and chronic mild stress paradigm	SD rats ICR mice	Recombinant human TFF3 (0.1 mg/kg, i.p.)	Mediates antidepressant-like effects via PI3K/AKT pathway.	[41]
Olfactory-bulbectomy-induced depression	SD rats	Recombinant human TFF3 (0.1 mg/kg, i.p.)	Mediates antidepressant-like effects via BDNF/ERK/CREB pathway.	[44]

*HPA* hypothalamic-pituitary-adrenal, *mTOR* mammalian target of rapamycin, *PI3K* phosphoinositide 3-kinase, *BDNF* brain-derived neurotrophic factor, *CREB* cyclic adenosine monophosphate response element binding protein.

### TFF3 in gastric ulcer

Gastric ulcer is the most common type of peptic ulcer, with an estimated lifetime prevalence of 5–10% in the general population [48]. Excessive gastric-acid secretion and weakened gastric mucosal protection are the main factors that cause peptic ulcer. Many factors contribute to the protection of mucosal surfaces throughout the gastrointestinal tract. For example, when peptic ulcer occurs, ulcer-associated cell lineage promotes ulcer restitution by secreting TFF peptides, epidermal growth factor, and transforming growth factor-α [49]. Detection of TFF3 expression is difficult in normal gastric mucosal tissues, as TFF3 tends to be expressed in the marginal area of the stomach, i.e., the cardia and antrum [50, 51]. However, TFF3 expression is enhanced in the gastric mucosa and periulcer tissues of patients and rats with gastric ulcer. A significant increase in TFF3 mRNA was detectable as early as day 2 after ulcer induction [52]; the level steadily increased at the ulcer margin after day 4 By 40 days, the TFF3 levels were elevated several hundred folds [53].

Animal studies have shown that TFF3 administration reduced gastric mucosal damage caused by alcohol, pylorus ligation, non-steroidal anti-inflammatory drugs, stress, and lipopolysaccharide (Table 4). Its effects include maintaining the gastric pH, stimulation of mucosal healing without proliferation [54], and local effects [55]. The protective effect is even more pronounced when mucus glycoproteins or epidermal growth factor are administered concomitantly [54, 56]. Unlike that of many gastroprotective agents, the gastroprotective effect of TFF3 is independent of prostaglandin (PG). This effect appears to be achieved at the mucosal luminal surface [55]. There are few studies on the gastroprotective effect of TFF3. Currently, it is only known that it may be achieved by activating the PI3K/AKT signaling pathway [57].

**Table. 4:** Protective effect of TFF3 on gastrointestinal tract.

Modeling method	Animal type	Source of TFF3	Dosage and mode of administration	Effectiveness and mechanism	Reference
Indomethacin- and restraint-induced gastric injury	SD rats	Recombinant rat TFF3 was produced using the vector pAX5	0.15 mg/kg, s.c.	TFF3 alone has no obvious effect, but its combined use with EGF induced 80% reduction in gastric damage.	[54]
Ethanol- and indomethacin-induced gastric injury	SD rats	Recombinant rat TFF3 produced from yeast	0–10 mg/kg, i.g.	Administration 2 h before injury significantly prevented gastric injury in a dose-dependent manner.	[55]
Ethanol-induced gastric injury	SD rats	TFF3 purified from human meconium	5 mg/kg, i.g.	Natural TFF3, abundant in the meconium, facilitates gastric mucosal protection.	[134]
	C57 mice Wistar rats	Murine TFF3 dimer was prepared at Novo Nordisk a/s	5 mg/kg, i.v. to mice 25 mg/kg, i.v. to rats	TFF3 administered systemically is absorbed by mucous neck cells in the stomach and secreted to the gastric luminal surface.	[135]
Gastric injury induced by water immersion restraint stress	SD rats	Recombinant rat TFF3	0.1 mg/kg, i.p.	Gastric mucosal protection through AKT signaling.	[57]
Hypoxia-induced necrotizing enterocolitis	Wistar rats	Recombinant human TFF3	0.5 mg/kg, i.p. 0.2 mg/kg, s.c.	There is no difference between intraperitoneal injection and subcutaneous injection of TFF3 for colitis treatment.	[136]
Mitomycin- or dextran-induced colitis	Wistar rats	Recombinant rat TFF3 produced from yeast	5 mg/kg, s.c. 5 mg/kg, luminally	Intracolonic administration of dimer, but not monomers, ameliorated colitis. Parenteral administration aggravated colitis, especially the TFF3 monomer.	[3]
Dextran-sulfate sodium-induced colitis	BALB/cAnNTac mice	Human and murine TFF3 dimer was prepared at Novo Nordisk A/S	1 and 25 mg/kg, i.p 1 mg/kg, s.c.	Different administration methods and TFF3 from different species produce slightly different effects.	[137]
Trinitrobenzene-sulfonic-acid-induced colitis	BALB/Cmice	Recombinant human TFF3	5 mg/kg, i.p.	Improves colitis by inhibiting TLR4/NF-κB signaling pathway.	[68]
Hypoxia/hypothermia-induced necrotizing enterocolitis	Wistar rats	Recombinant human TFF3	0.2 mg/kg, s.c.	Improved necrotizing enterocolitis by inhibiting inflammation.	[69]

*EGF* epidermal growth factor, *TLR* toll-like receptor, *NF-κB* nuclear factor κB.

### TFF3 in colitis

The intestinal epithelium is the main barrier that protects us from bacteria and immunogenic or toxic luminal contents. Intestinal barrier dysfunction favors aberrant antigen uptake and immune-cell infiltration, triggering uncontrolled inflammation that progresses to colitis. Intestinal goblet cells can secrete TFF3 and mucin2 (MUC2), and they are bound in soluble portions of the intestinal mucus by covalent interactions to form heteropolymers, which in turn constitute the first line of defense at the intestinal barrier [58]. TFF3^−/−^ mice showed in increased colonic mucosal permeability and sensitivity to dextran sulfate sodium, as well as poor epithelial regeneration [15, 59]. Different administration methods for TFF3 dimer significantly ameliorated the severity of colitis induced by dextran sodium sulfate, radiochemotherapy, hypoxia, and trinitrobenzene sulfonic acid (Table 4). Notably, a detailed comparative study demonstrated that intraluminal administration was more effective than systemic administration [3]. Subcutaneous injections and especially administration of TFF3 monomers aggravate colitis induced by mitomycin C or dextran sulfate sodium salt [3]. Moreover, the monomeric and dimeric forms of TFF3 are functionally different. The pro-proliferative and anti-apoptotic functions of TFF3 have been reported to require homodimerization [3, 4, 60], whereas exerting a pro-migratory effect does not require an intact trefoil domain or dimerization [4].

TFF3 rapid responds to injury, and it can reduce intestinal epithelial permeability by regulating tight junctions [61–63], an effect that can be attenuated by inhibition of the PI3K/AKT signaling pathway [64]. In addition, the process of restitution requires TFF3 to act as a motogen, by promoting epithelial cell elongation and migration to cover the exfoliated surface. Studies have shown that TFF3 treatment enhances the collective migration of IEC-18 cells and forms continuous sheets of migrating cells to ensure precise coverage of the re-populated area [65]. The number of migrating cells after treatment with ERK and Janus kinase (JAK) inhibitors was lower than that after treatment with either inhibitor alone [66]. It is suggested that TFF3 may promote intestinal mucosal reconstitution through crosstalk between ERK and JAK/signal transducer and activator of transcription (STAT3) pathways (as shown in Fig. 1C).

Inhibition of inflammatory response is an important mechanism through which TFF3 ameliorates colitis. Because of the destruction of intestinal barrier, activated leukocytes are recruited to the lamina propria, and they secrete a large number of cytokines, which is the key event causing intestinal mucositis. TFF3 has been shown to be regulated by cytokines and toll-like receptor (TLR) signaling. TLR2 ligands can stimulate intestinal goblet cells to selectively synthesize a large amount of TFF3. Similarly, TLR2-deficient mice showed innate immune deficiency of goblet-cell-derived TFF3 [67]. TFF3 and TLR2 are functionally linked, and overexpression of both blocked IL1β-induced upregulation of proinflammatory cytokines via the PI3K/AKT pathway [64]. Nuclear factor κB (NF-κB) is considered the main regulator of intestinal inflammatory response. TFF3 inhibits NF-κB activity via the TLR4 and ERK/twist signaling pathways, thereby attenuating the inflammatory reaction of IEC-18 cells and mouse intestines [68–72]. Moreover, the addition of TFF3 isolated from human milk to HT29 cells inhibited LPS-induced IL-6 and IL-8 secretion, a process dependent on protease-activated receptor 2 (PAR-2) [73, 74]. Interestingly, leucine-rich repeat receptor and nogo-interacting protein 2 (LINGO2) was detected in immune cells, including CD4^+^T cells, myeloid cells, and B cells [75]. LINGO2 was confirmed to capture TFF3 at 40 nm of the epithelial cell membrane. The TFF3–LINGO2 interaction led to enhanced epidermal growth factor receptor (EGFR) activation, which promoted wound healing and immunity [75]. Therefore, it is highly worth investigating whether TFF3 interacts with the LINGO2 of immune cells to regulate intestinal inflammation.

### TFF3 in cancer

**Fig. 2:**
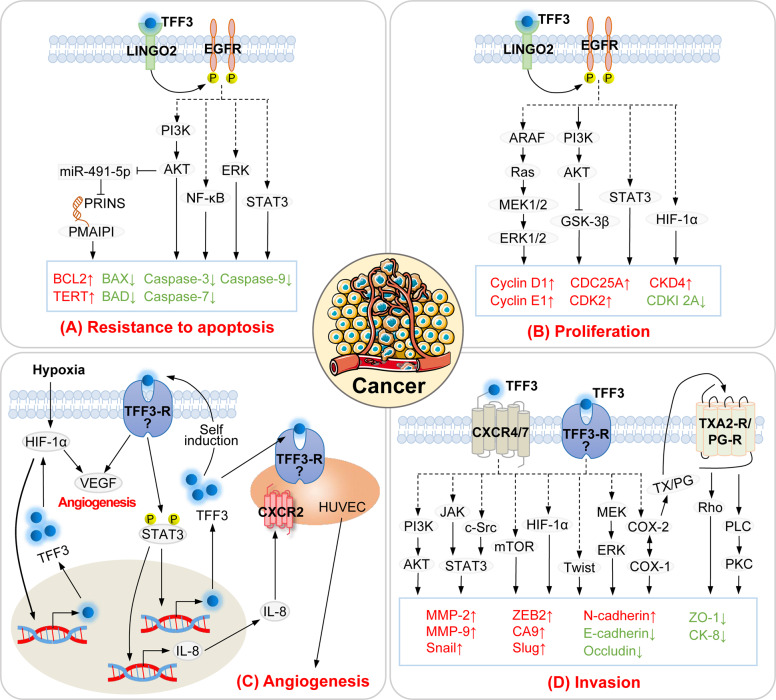
Carcinogenic mechanism of TFF3. TFF3 affects apoptosis (**A**) and cell cycle progression (**B**) by activating MAPK, NF-κB, PI3K, STAT3, and HIF-1α signaling pathways. TFF3 does not bind to EGFR, but it can induce its phosphorylation. Treatment with the specific EGFR inhibitors tyrphostin A25 and AG1478 can significantly inhibit TFF3-induced cell survival and proliferation. **C** The promoter region of TFF3 contains the binding sites of HIF-1α and STAT3, which enables TFF3 to achieve self-induction. On the one hand, TFF3 enhanced the induction of VEGF by HIF-1α; on the other hand, it induced IL-8 expression via STAT3 to promote angiogenesis. **D** TFF3 regulates the expression of proteins related to cell migration by activating the MAPK, NF-κB, PI3K, STAT3, mTOR, and HIF-1α signaling pathways. CXCR, TX2A-R, and PG-R are involved in this biological effect of TFF3. Although TFF3 does not directly bind to TXA2-R and PG-R, it may activate them by increasing the expression of COX or COX derivatives such as prostaglandin E(2) and prostaglandin I(2). The combination of TFF3 and CXCR4/7 can induce cell migration; however, the signaling pathway underlying this effect remains to be verified. Established functions are denoted by solid arrows, whereas unidentified pathways are indicated by question marks. PRINS: psoriasis susceptibility-related RNA gene induced by stress; PMAIP1: phorbol-12-myristate-13-acetate-induced protein 1; TERT, telomerase reverse transcriptase; LINGO2, leucine-rich repeat receptor and nogo interacting protein 2; PI3K, phosphoinositide 3-kinase; NF-κB, nuclear factor κB; EGFR, epidermal growth factor receptor; ERK, extracellular signal-regulated kinase; STAT:, signal transducer and activator of transcription; GSK-3β, glycogen synthase kinase 3 beta; HIF, hypoxia-inducible factor; CDC, cell division cycle; CKDI, cyclin-dependent kinase inhibitor; VEGF, vascular endothelial growth factor; IL, Interleukins; CXCR, C-X-C chemokine receptor; HUVEC, human umbilical vein endothelial cells, mTOR, mammalian target of rapamycin; COX, cyclooxygenase; TXA2-R, thromboxane A2 receptor; PG-R: prostaglandin receptor; MMP, matrix metalloproteinase; ZEB2, zinc finger E-box-binding homeobox 2; CA9, carbonic anhydrase IX; ZO-1, zonula occludens-1; CK-8, cytokeratin-8; PLC, phospholipase C; PKC, protein kinase C.

The GLOBOCAN reports almost 10 million deaths from cancer in 2020, rendering cancer a leading cause of death worldwide [76]. Emerging evidence suggests that TFF3 is significantly upregulated in cancers such as gastric cancer, colorectal cancer, lung cancer, thyroid cancer, and breast cancer and that it plays a key role in tumor progression [77]. The occurrence and development of cancer mainly involves the proliferation, metastasis, angiogenesis, and resistance to apoptosis of tumor cells. TFF3 is involved in these four important carcinogenic processes through mitogen-activated protein kinase (MAPK)/ERK, PI3K/AKT, STAT3, and hypoxia-inducible factor (HIF)-1α signaling pathways (Fig. 2 and Supplementary Tables 1, 2 and 3). For example, the use of siRNA to silence TFF3 in cancer cells inhibited of MAPK/ERK signaling pathway [78, 79], leading to a significant decrease in cell survival, whereas exogenous administration of TFF3 significantly activated the ERK pathway and promoted cancer-cell invasion [4, 80]. Hypoxia is an early signal for induction of the tumor angiogenesis switch. It time-dependently induced TFF3 expression in colonic, gastric, and renal tubular epithelial cells [81–83]. The promoter region of TFF3 contains binding sites for HIF-1α; blocking HIF-1α expression substantially decreased TFF3 mRNA expression over 4 and 24 h of hypoxia [81]. TFF3 overexpression time-dependently upregulated hypoxia-induced vascular endothelial growth factor and HIF-1α mRNA expression [84]. These results suggested that TFF3 may be mutually induced with HIF-1α, promoting angiogenesis. Furthermore, the promoter region of TFF3 contains binding sites for STAT3, which enables self-induction of TFF3 [85]. STAT3 inhibition has been consistently shown to hinder the proliferation, invasion, and survival of TFF3-induced cancer cells [66, 86–89].

A novel specific small-molecule TFF3 inhibitor, AMPC, disrupted dimerization with TFF3 by exploiting the cysteine-57 residue essential for TFF3 homodimerization. Since monomeric TFF3 is characterized by faster degradation than dimeric TFF3, inhibiting its homodimerization could promote TFF3 depletion [90]. Studies showed that AMPC dose-dependently decreased TFF3 expression in estrogen-receptor-positive mammary carcinoma cells, lung adenocarcinoma cells, and mesenchymal colorectal carcinoma cells, which induced tumor-volume reduction in mice [78, 91, 92]. AMPC in combination with anticancer agents (5-fluorouracil or doxorubicin) produced synergistic tumor-suppressive effects. In the future, TFF3 may be used as a molecular target of functional antagonism in combination with other chemotherapeutic drugs to slow down cancer progression.

## Functional proteins and receptors of TFF3

### MUC2, FCGBP, and DMBT1

Although MUC2, IgG Fc-binding protein (FCGBP), and Deleted in malignant brain tumor 1 (DMBT1) are TFF3-binding partners that were identified early on, none of them appear to possess the typical receptor characteristics, and there is no evidence that their binding can transmit signals to cells. MUC is a major component of the mucus layer covering the intestinal surface, which sequesters gut microbes from contact with host epithelial and immune cells and plays an important role in maintaining mucosal homeostasis. MUC are closely related to TFFs because they are usually secreted by goblet cells together. Each member of the TFF family is co-localized with the designated MUC type, for example, TFF1 with MUC5AC, TFF2 with MUC6, and TFF3 with MUC2 [49, 93, 94]. Studies in intestinal epithelial cell restitution models have found that although TFFs alone can increase the migration rate of cells to the wound site by 3–6 times, the combined use of TFFs and MUC can considerably increase the rate up to 15 times [56].

FCGBP, the Fc portion of the IgG-binding site in the intestinal epithelium, is an important component of the mucosal immune defense [95]. FCGBP in the intestine and saliva is the disulfide linkage partner protein of TFF3 [96, 97]. TFF3 can be released from purified TFF3–FCGBP heteromeric complexes in vitro by hydrogen sulfide reduction. This mechanism is consistent with the high H_2_S concentration in disease tissue. Therefore, this function can be used as a reservoir for biologically active peptides. Currently, it is unclear which signaling pathways and molecular functions are activated after TFF3 binds to FCGBP. TFF3–FCGBP complexes may play a role in innate immune defense of mucous epithelial cells such as oral cavity [97, 98].

DMBT-1 is a gene encoding an alternatively spliced protein involved in epithelial regeneration and innate host defense and is significantly upregulated in inflammatory bowel disease [60]. It is a pattern recognition receptor with multiple binding sites to which TFF3 can bind in a calcium-dependent manner. Till date, the role of the TFF3–DMBT1 heterodimer has not been clarified. Considering that animal models lacking DMBT1 and TFF proteins confer increased susceptibility to inflammatory bowel disease, scholars speculate that the DMBT1–TFF3 interaction may play a role in normal gastrointestinal homeostasis and inflammatory bowel disease [60].

### TXA2-R and PG-R

Thromboxane A2 receptor (TXA2-R) and PG-R may be indirect acting receptors for the pro-invasive role of TFF3. Cyclooxygenases (COX) produce TXA2 and various PGs, which promote cell invasion through the TXA2-R and PG-R, respectively [99, 100]. Studies showed that TFF3 enhanced epithelial cell invasion in a TXA2-R- and COX-dependent manner; this behavior was abrogated by treatment with inhibitors of COX, TXA2-R, Rho, phospholipase C, protein kinase C, PI3K, and mTOR alone [101, 102]. Although TFF3 does not directly bind to PG-R and TXA2-R, it may activate PG-R/TXA2-R by increasing the expression of COX [103] or COX derivatives such as PGE(2) and PGI(2) [104]. Furthermore, Rodrigues et al. discussed the possibility that the COX-derived products TXA2 and PGH(2) are downstream effectors of TFF-induced cell invasion [102].

### PAR-2 and CXCR4/7

For a long time, the search for TFF3-binding proteins with typical receptor characteristics did not made great progress. It was not until 2016 that TFF3 was identified as a low affinity ligand for C-X-C chemokine receptor (CXCR) 4, CXCR7, and PAR-2. PAR-2, which belong to the G-protein-coupled receptor family members, exhibit broad physiological functions and activate various cellular transcription factors by mediating the ERK1/2 signaling pathway. Using western blotting and immunoprecipitation, researchers demonstrated the interaction between TFF3 and PAR-2, which induced the expression of human beta defensins and suppressed the levels of the cytokines IL-6 and IL-8 in intestinal epithelial cells [74].

CXCR is widely expressed in various cells and tissues and involved in cell migration, immune cell recruitment, hematopoietic function, embryonic development, and tumor metastasis. Several studies have indicated that MAPK is the downstream pathway of CXCR4/7. Co-expression of CXCR4/7 enhanced the ERK1/2 and p38 MAPK signaling pathways [105]. Interestingly, the addition of a specific CXC receptor antagonist completely inhibited the enhanced migration of conjunctival epithelial cells stimulated by TFF3; however, the MAPK signal was not affected by the inhibition of CXCR4/7 [106]. This indicated that TFF3 induced intracellular MAPK through other binding targets, such as EGFR, as discussed below.

### EGFR and LINGO2

EGFR is a membrane surface receptor closely related to the growth, proliferation, differentiation, apoptosis, metastasis, and other processes of tumor cells. Till date, no studies have shown that TFF3 directly binds to EGFR. They may interact indirectly. Nevertheless, TFF3 has the ability to cause rapid tyrosine phosphorylation of EGFR [107] and subsequent activation of downstream MAPK pathways [80, 108]. In turn, the specific inhibition of EGFR blocked the differentiation and proliferation of cells promoted by TFF3 [109, 110].

LINGO2 belongs to the family of leucine-rich repeat and IgG-like domain proteins with a large extracellular portion (ectodomain), a transmembrane domain, and a short cytoplasmic tail. LINGO2-related genetic variants are associated with obesity, waist circumference, and BMI in different populations [111–113]; they might act as susceptibility genes for gestational diabetes mellitus [114]. Recent studies identified LINGO2 as a key component of TFF3 cellular reactivity, and their binding was observed within 40 nm of the cell membrane of intestinal epithelial cells. The TFF3–LINGO2 interaction resulted in enhanced EGFR signaling, which could promote wound healing and immunity [75]. However, this may lead to carcinogenic behavior; tissue microarray analysis revealed that LINGO2 expression was significantly increased in advanced gastric cancer, and the overall survival rate of patients with high LINGO2 was significantly lower than that of patients with low LINGO2 [115]. Corresponding to the carcinogenic characteristics of TFF3, we speculate that the enhanced cell motility, angiogenesis, and tumorigenicity observed in LINGO2-overexpressing cells [115] are likely directly related to TFF3.

## Clinical applications of TFF-peptide-based therapies

Currently, only recombination TFF3 has entered clinical practice; it is used for treating ulcerative colitis and oral mucositis (Table 5) and produced by recombinant expression in yeast. Despite the crucial role of TFF3 for gastrointestinal mucosal protection, as demonstrated at the cellular and animal level, these models do not represent the human situation well. No positive effects of TFF3 were observed in a phase I/II double-blind randomized trial of TFF3 enema combined with oral 5-aminosalicylic acid in the treatment of ulcerative colitis [116]. Compared with increasing the oral dose of mesalazine, TFF3 enema showed no other advantages. The poor effect can be attributed to the insufficient number of cases and route of administration. For example, gavage of colitis mice with TFF1-overexpressing *Lactococcus lactis* achieved much greater efficacy than oral or rectal administration of pure recombinant TFF1 [117]. Furthermore, intraluminal administration of dimeric TFF3 significantly improved colitis scores in a colitis model. However, systemic administration, especially TFF3 monomer, aggravated colitis severity in rats [3]. These results suggested that different administration pathways have a great impact on TFF peptide function.

**Table. 5:** Clinical trial study of drugs developed for TFF peptides.

Year	Disease	Investigational medicinal product	Clinical stage	Recruitment numbers	Dosage	Effectiveness	Reference
2013	Chemotherapy-induced oral mucositis	AG013, an oral rinse composed of recombinant L. lactis strain that secretes mucosal protectant hTFF1	Phase 1b	25	15 mL	AG013 was safe and well tolerated, but 96% of subjects experienced at least one adverse event. The most common adverse effect was nausea (11 of 25, 44%).	[138]
2009	Chemotherapy-induced oral mucositis	Recombinant human TFF3 oral spray	Phase II	99	10 mg/mL; 80 mg/mL	It effectively reduced the incidence and severity of oral mucositis; only 6.1% patients experienced adverse events.	[118]
2005	Mild-to-moderate left-sided ulcerative colitis	Enema prepared using Recombinant human TFF3 enema	phase I/II	16	10 mg/mL	Well tolerated, but no TFF3-associated positive effect was observed.	[116]

Initial success has been achieved with the application of TFF peptides in the form of gargle and aerosol. Peterson *et al*. observed that prophylactic administration of TFF3 aerosol reduced the incidence and severity of clinically significant oral mucositis in a cohort of colorectal cancer patients [118]. Therefore, TFF3 not only exerts its effect on the digestive system but also served as a promising treatment for mucosal inflammation in various tissues, such as the ocular tissue [119], oral cavity [97, 120], and respiratory tract [121].

## Conclusions and future perspectives

TFF3 plays a therapeutic role in a variety of diseases. As a mixed ligand, TFF3 can activate multiple signaling pathways including MAPK, NF-κB, PI3K, STAT3, mTOR, and HIF-1α to repair damaged mucosa, regulate glucose and lipid metabolism; and produce anti-neuroinflammatory, antidepressant, and anti-drug-addictive properties. However, TFF3 is the driving factor of cancer, which is involved in the proliferation, invasion, resistance to apoptosis, and angiogenesis of cancer cells. There needs to be a good balance between its excellent mucosal restitution ability and its carcinogenic function. More in-depth studies are urgently needed to determine the balance point for elucidating the factors that determine the carcinogenic function of TFF3. Moreover, there remain major gaps in the knowledge of TFF3-specific receptors, molecular mechanisms, drug-delivery strategies, and physiological significance. These problems are an important reason that prevented TFF3 from being used as a therapeutic target in clinic.

Considering the diverse biological functions of TFF3, mucosal protection would not be the only application of value. For example, TFF3 dimer in the tear film and ocular surface is very promising as a therapeutic agent for dry eye syndrome [119, 122]. TFF3 is expected to be a potential therapeutic target for glucose- and lipid-metabolism diseases since it can affect hepatic fatty acid accumulation [19], stimulate pancreatic β-cell proliferation [110], and improve insulin sensitivity [28]. TFF3–FCGBP may be a component of new antimicrobial agents, such as for use as artificial saliva. TFF3 exhibits significant neurologic protection after injection of recombinant TFF3 intraperitoneally or in the basolateral amygdala. The combined use of a TFF3 inhibitor with chemotherapeutic drugs exhibited synergistic tumor-suppressive effects, and it may serve as a potential therapy for slowing cancer progression in the future. Further exploration is needed to determine whether TFF3 can interact with LINGO2 of immune cells to participate in intestinal immune response. As the understanding of TFF3 biological function accelerates, its use in clinical medicine can be expected to increase, such as for dry eye disease, neurodegeneration, glycolipid-metabolism disorders, cancer, and immune imbalance. Its potential value for clinical diagnosis will be further explored and demonstrated.

## Supplementary information

Supplementary Table

Reproducibility Checklist

Detailed Author Contribution form

## Data Availability

Data sharing is not applicable to this article as no datasets were generated or analyzed during the current study.
