# Supplementary material for: Pathological and therapeutic roles of bioactive peptide trefoil factor 3 in diverse diseases: recent progress and perspective
Source: Cell Death Dis. 2022 Jan 17;13(1):62. doi: 10.1038/s41419-022-04504-6 (PMC8763889; doi:10.1038/s41419-022-04504-6)
Supplement: Supplementary file 1 — Supplementary Table [file 41419_2022_4504_MOESM1_ESM.doc]

**Supplementary materials**

**Supplementary Table. 1 Mechanisms of TFF3 in promoting cell migration and invasion.**

| **Cell line** | **Intervention methods (dosage)** | **Mechanism** | **Reference** |
| --- | --- | --- | --- |
| HT29 | Addition of recombinant TFF3 (10 mM) | Leads to rapid and specific tyrosine phosphorylation of β-catenin and epidermal growth factor receptors in cells | 1 |
| HT29, HCT116, SW480 | Addition of recombinant TFF3 (10^−8^ M and 10^−9^ M) | Disrupts the complex between E-cadherin, β-catenin, and associated proteins. | 2 |
| IEC-6 | Addition of TFF3 fusion proteins (3 mg/mL) | Promotes MEK/ERK pathway activation. | 3 |
| HEK293, MDCK, HT29/B6 | Plasmid transfection | Modulation of E-cadherin-mediated cell–cell contacts. | 4 |
| Oral keratinocytes, oral cancer cell line, D12 | Addition of recombinant TFF3 dimer (0.25 and 0.5 μM) | Activates ERK1/2 pathway. | 5 |
| GES-1 | Addition of recombinant TFF3 (100 and 500 ng/ml) | Activates PI3K/AKT pathway. | 6 |
| HEK293 | Infection with recombinant adenovirus; addition of recombinant TFF3 (60 μg/mL) | Induce crosstalk between the ERK and JAK/STAT3 pathways. | 7 |
| SGC7901 | Plasmid transfection | Activates the Twist-dependent pathway | 8 |
| Human corneal epithelial cells | Addition of recombinant TFF3 (10, 30, 100, 300, 1000 μg/mL) | CXCR4/7 is involved in TFF3-dependent activation of migration. ERK1/2 pathway is activated in the process but not influenced by CXCR4/7. | 9 |
| MDCKT23, HCT-8/S11, HCT-8/S11/R1, PC/AA/C1 | Addition of recombinant TFF3 (0.1 μM) | Depends on PI3K, PLC, PKC, and mTOR signaling pathways. | 10 |
| MDCKts.src | Addition of recombinant TFF3 (0.1 μM) | Depends on COX and TXA2-R signaling pathways. | 11 |
| Rat-2 | Plasmid transfection | Regulation of invasion-related gene expression. | 12 |
| MDCKts.src | Addition of recombinant TFF3 (0.1 μM) | TFF3 pro-invasive signaling pathways are EGFR-independent. | 13 |
| HT29, SW620, LoVo | Plasmid transfection | Activates EMT progression. | 14 |
| TPC-1 | Plasmid transfection | Promotes EMT process through the MAPK/ERK pathway. | 15 |
| SiHa, Hela | Plasmid transfection | Activates the STAT3/E-cadherin signaling pathway. | 16 |
| TPC-1 | Plasmid transfection | Activates the PI3K/AKT pathway and decreases MMP-9 expression. | 17 |
| HCT8/S11  HEK-293T | Plasmid transfection; addition of recombinant TFF3 (0.1 μmol/L) | Activate STAT3 signaling through Tyr705 phosphorylation of both STAT3α and STAT3β isoforms. | 18 |
| MCF7, T47D | Plasmid transfection | Inhibits E-cadherin via c-SRC/STAT3 signaling pathway. | 19 |
| U87, U251 | Plasmid transfection | Promotes cell migration and invasion by regulating HIF-1α. | 20 |

**Supplementary Table. 2 Mechanism of TFF3 resistance to apoptosis.**

| **Cell line** | **Intervention methods (dosage)** | **Mechanism** | **Reference** |
| --- | --- | --- | --- |
| IEC-18 | Addition of recombinant TFF3 (6.25 μM) | Activates the NF-κB signaling pathway. | 21 |
| HCT116, IEC-6 | Addition of TFF3 fusion proteins (3 mg/mL) | Activates the EGFR and PI3K/AKT signaling pathways, which require intact dimerized peptide. | 22 |
| HT-29, HCT116, AGS, IEC-6 | Plasmid transfection; addition of recombinant TFF3 (1 mg/mL) | Activate EGFR and PI3K signaling pathways. | 23 |
| LNCap.FGC, PC-3, WPMY-1 | Plasmid transfection | Blocks mitochondrial-induced apoptosis by activating the PI3K/AKT pathway. | 24 |
| HT-29/B6 | Plasmid transfection | Mediates miR-491-5p down-regulation and PRINS accumulation through PI3K/AKT pathway | 25 |
| Hep3B, Huh7 | Plasmid transfection | Regulates Bcl-2 expression by activating AKT. | 26 |
| Caco2 | Plasmid transfection | Activates the ERK1/2 pathway. | 27 |
| TPC-1 | Plasmid transfection | Activates the PI3K/AKT pathway and decreased BCL-2 expression. | 28 |

**Supplementary Table. 3 Mechanisms of TFF3 in promoting cell proliferation.**

| **Cell line** | **Intervention methods** | **Mechanism** | **Reference** |
| --- | --- | --- | --- |
| MCF10A, MCF12A, HMEC-hTERT | Plasmid transfection | Utilizes STAT3 activity to promote cell proliferation. | 29 |
| 832/13 | Plasmid transfection | Utilizes AKT and EGFR activity to promote cell proliferation. | 30 |
| HT29 | siRNA transfection | Utilizes EGFR/AKT/GSK-3β pathway to promote cell proliferation. | 31 |
| U87, U251 | Plasmid transfection | Utilizes a hypoxia-independent HIF-1α induction to drive abnormal cell proliferation | 32 |
| H1299, H1975 | Plasmid transfection | Activates MAPK/ERK pathway by increasing ARAF expression. | 33 |

**Reference：**

1. Liu D, el-Hariry I, Karayiannakis A, Wilding J, Chinery R, Kmiot W*, et al.* Phosphorylation of beta-catenin and epidermal growth factor receptor by intestinal trefoil factor. *Laboratory investigation; a journal of technical methods and pathology* 1997, **77**(6)**:** 557-563.

2. Efstathiou J, Noda M, Rowan A, Dixon C, Chinery R, Jawhari A*, et al.* Intestinal trefoil factor controls the expression of the adenomatous polyposis coli-catenin and the E-cadherin-catenin complexes in human colon carcinoma cells. *Proceedings of the National Academy of Sciences of the United States of America* 1998, **95**(6)**:** 3122-3127.

3. Kinoshita K, Taupin D, Itoh H, Podolsky D. Distinct pathways of cell migration and antiapoptotic response to epithelial injury: structure-function analysis of human intestinal trefoil factor. *Molecular and cellular biology* 2000, **20**(13)**:** 4680-4690.

4. Meyer zum Büschenfelde D, Hoschützky H, Tauber R, Huber O. Molecular mechanisms involved in TFF3 peptide-mediated modulation of the E-cadherin/catenin cell adhesion complex. *Peptides* 2004, **25**(5)**:** 873-883.

5. Storesund T, Hayashi K, Kolltveit K, Bryne M, Schenck K. Salivary trefoil factor 3 enhances migration of oral keratinocytes. *European journal of oral sciences* 2008, **116**(2)**:** 135-140.

6. Sun Z, Liu H, Yang Z, Shao D, Zhang W, Ren Y*, et al.* Intestinal trefoil factor activates the PI3K/Akt signaling pathway to protect gastric mucosal epithelium from damage. *International journal of oncology* 2014, **45**(3)**:** 1123-1132.

7. Le J, Zhang D, Zhao Y, Qiu W, Wang P, Sun Y. ITF promotes migration of intestinal epithelial cells through crosstalk between the ERK and JAK/STAT3 pathways. *Scientific reports* 2016, **6:** 33014.

8. Zheng Q, Gao J, Li H, Guo W, Mao Q, Gao E*, et al.* Trefoil factor 3 peptide regulates migration via a Twist-dependent pathway in gastric cell. *Biochemical and biophysical research communications* 2013, **438**(1)**:** 6-12.

9. Dieckow J, Brandt W, Hattermann K, Schob S, Schulze U, Mentlein R*, et al.* CXCR4 and CXCR7 Mediate TFF3-Induced Cell Migration Independently From the ERK1/2 Signaling Pathway. *Investigative ophthalmology & visual science* 2016, **57**(1)**:** 56-65.

10. Emami S, Le Floch N, Bruyneel E, Thim L, May F, Westley B*, et al.* Induction of scattering and cellular invasion by trefoil peptides in src- and RhoA-transformed kidney and colonic epithelial cells. *FASEB journal : official publication of the Federation of American Societies for Experimental Biology* 2001, **15**(2)**:** 351-361.

11. Rodrigues S, Nguyen Q, Faivre S, Bruyneel E, Thim L, Westley B*, et al.* Activation of cellular invasion by trefoil peptides and src is mediated by cyclooxygenase- and thromboxane A2 receptor-dependent signaling pathways. *FASEB journal : official publication of the Federation of American Societies for Experimental Biology* 2001, **15**(9)**:** 1517-1528.

12. Chan VY, Chan MW, Leung WK, Leung PS, Sung JJ, Chan FK. Intestinal trefoil factor promotes invasion in non-tumorigenic Rat-2 fibroblast cell. *Regul Pept* 2005, **127**(1-3)**:** 87-94.

13. Rodrigues S, Attoub S, Nguyen Q, Bruyneel E, Rodrigue C, Westley B*, et al.* Selective abrogation of the proinvasive activity of the trefoil peptides pS2 and spasmolytic polypeptide by disruption of the EGF receptor signaling pathways in kidney and colonic cancer cells. *Oncogene* 2003, **22**(29)**:** 4488-4497.

14. Yusufu A, Shayimu P, Tuerdi R, Fang C, Wang F, Wang H. TFF3 and TFF1 expression levels are elevated in colorectal cancer and promote the malignant behavior of colon cancer by activating the EMT process. *International journal of oncology* 2019, **55**(4)**:** 789-804.

15. Lin X, Zhang H, Dai J, Zhang W, Zhang J, Xue G*, et al.* TFF3 Contributes to Epithelial-Mesenchymal Transition (EMT) in Papillary Thyroid Carcinoma Cells via the MAPK/ERK Signaling Pathway. *Journal of Cancer* 2018, **9**(23)**:** 4430-4439.

16. Yuan Z, Chen D, Chen X, Yang H, Wei Y. Overexpression of trefoil factor 3 (TFF3) contributes to the malignant progression in cervical cancer cells. *Cancer cell international* 2017, **17:** 7.

17. Wu J, Zhang H, Zhang W, Zhang J, Lin X, Xue G*, et al.* Lentivirus-mediated shRNA interference of trefoil factor 3 blocks cell viability, migration and invasion in the papillary thyroid carcinoma cells. *Neoplasma* 2018, **65**(2)**:** 169-177.

18. Rivat C, Christine R, Rodrigues S, Sylvie R, Bruyneel E, Erik B*, et al.* Implication of STAT3 signaling in human colonic cancer cells during intestinal trefoil factor 3 (TFF3) -- and vascular endothelial growth factor-mediated cellular invasion and tumor growth. *Cancer research* 2005, **65**(1)**:** 195-202.

19. Pandey V, Wu Z, Zhang M, Li R, Zhang J, Zhu T*, et al.* Trefoil factor 3 promotes metastatic seeding and predicts poor survival outcome of patients with mammary carcinoma. *Breast cancer research : BCR* 2014, **16**(5)**:** 429.

20. Diao S, Zheng Q, Gao J, Yao Y, Ren S, Liu Y*, et al.* Trefoil factor 3 contributes to the malignancy of glioma via regulating HIF-1α. *Oncotarget* 2017, **8**(44)**:** 76770-76782.

21. Chen Y, Lu Y, De Plaen I, Wang L, Tan X. Transcription factor NF-kappaB signals antianoikic function of trefoil factor 3 on intestinal epithelial cells. *Biochemical and biophysical research communications* 2000, **274**(3)**:** 576-582.

22. Kinoshita K, Taupin D, Itoh H, Podolsky D. Distinct pathways of cell migration and antiapoptotic response to epithelial injury: structure-function analysis of human intestinal trefoil factor. *Molecular and cellular biology* 2000, **20**(13)**:** 4680-4690.

23. Taupin D, Kinoshita K, Podolsky D. Intestinal trefoil factor confers colonic epithelial resistance to apoptosis. *Proceedings of the National Academy of Sciences of the United States of America* 2000, **97**(2)**:** 799-804.

24. Liu J, Kim S, Shin S, Jung S, Yim S, Lee J*, et al.* Overexpression of TFF3 is involved in prostate carcinogenesis via blocking mitochondria-mediated apoptosis. *Experimental & molecular medicine* 2018, **50**(8)**:** 1-11.

25. Hanisch C, Sharbati J, Kutz-Lohroff B, Huber O, Einspanier R, Sharbati S. TFF3-dependent resistance of human colorectal adenocarcinoma cells HT-29/B6 to apoptosis is mediated by miR-491-5p regulation of lncRNA PRINS. *Cell death discovery* 2017, **3:** 16106.

26. You M, Chen Y, Chong Q, Wu M, Pandey V, Chen R*, et al.* Trefoil factor 3 mediation of oncogenicity and chemoresistance in hepatocellular carcinoma is AKT-BCL-2 dependent. *Oncotarget* 2017, **8**(24)**:** 39323-39344.

27. Chen R, Chiou Y, Chong Q, Poh H, Tan T, Zhang M*, et al.* Pharmacological Inhibition of TFF3 Enhances Sensitivity of CMS4 Colorectal Carcinoma to 5-Fluorouracil through Inhibition of p44/42 MAPK. *International journal of molecular sciences* 2019, **20**(24).

28. Wu J, Zhang H, Zhang W, Zhang J, Lin X, Xue G*, et al.* Lentivirus-mediated shRNA interference of trefoil factor 3 blocks cell viability, migration and invasion in the papillary thyroid carcinoma cells. *Neoplasma* 2018, **65**(2)**:** 169-177.

29. Pandey V, Zhang M, You M, Zhang W, Chen R, Zhang W*, et al.* Expression of two non-mutated genetic elements is sufficient to stimulate oncogenic transformation of human mammary epithelial cells. *Cell death & disease* 2018, **9**(12)**:** 1147.

30. Fueger P, Schisler J, Lu D, Babu D, Mirmira R, Newgard C*, et al.* Trefoil factor 3 stimulates human and rodent pancreatic islet beta-cell replication with retention of function. *Molecular endocrinology (Baltimore, Md)* 2008, **22**(5)**:** 1251-1259.

31. Raja S, Murali M, Devaraj H, Devaraj S. Differential expression of gastric MUC5AC in colonic epithelial cells: TFF3-wired IL1 β/Akt crosstalk-induced mucosal immune response against Shigella dysenteriae infection. *Journal of cell science* 2012, **125:** 703-713.

32. Diao S, Zheng Q, Gao J, Yao Y, Ren S, Liu Y*, et al.* Trefoil factor 3 contributes to the malignancy of glioma via regulating HIF-1α. *Oncotarget* 2017, **8**(44)**:** 76770-76782.

33. Zhang M, Wang B, Chong Q, Pandey V, Guo Z, Chen R*, et al.* A novel small-molecule inhibitor of trefoil factor 3 (TFF3) potentiates MEK1/2 inhibition in lung adenocarcinoma. *Oncogenesis* 2019, **8**(11)**:** 65.
