# Supplementary material for: Pathological and therapeutic roles of bioactive peptide trefoil factor 3 in diverse diseases: recent progress and perspective
Source: Cell Death Dis. 2022 Jan 17;13(1):62. doi: 10.1038/s41419-022-04504-6 (PMC8763889; doi:10.1038/s41419-022-04504-6)
Supplement: Supplementary file 3 — Detailed Author Contribution form [file 41419_2022_4504_MOESM3_ESM.pdf]

DECLARATION OF CONTRIBUTIONS TO ARTICLE

ADMC

Manuscript Number: #CDDIS-21-2931R	Journal Name: *Cell Death & Disease*	(the 'Journal')
Proposed Title of the Contribution: Pathological and Therapeutic Roles of Bioactive Peptide Trefoil Factor 3 in Diverse Diseases: Recent Progress and Perspective		(the 'Contribution)
Author(s): Yiqi Yang, Ziyang Lin, Quanyou Lin, Weijian Bei, Jiao Guo		(the 'Authors')

For all *CDDis* articles, each person named as an author in the published version must be able to show he or she has contributed substantially to the article.

Authorship credit should be based on 1) substantial contributions to conception and design, acquisition of data, or analysis and interpretation of data; 2) drafting the article or revising it critically for important intellectual content; and 3) final approval of the version to be published. Authors should meet conditions 1, 2 and 3.

Any person who cannot be shown to have made a substantial contribution to the article cannot be listed as an author in the final version. The name of any person who is deemed to have made a minor contribution can, however, appear in the Acknowledgments section of the article.

Please complete the table below to indicate the contributions of all named authors to the manuscript.

Author Full Name:	Specification of Contribution to the Manuscript:
Yiqi Yang	Contribute to concept, direction, framework design and article revision.
Ziyang Lin	Substantial contributions to article writing, drawing, tabulation and data acquisition.
Quanyou Lin	Contribute to reference checking, data acquistion and the writing of some chapters.
Beiwei Jian	Contribute to critical revision of important intellectual content.
Jiao Guo	Contribute to concept, direction, critical revision and final approval of the version to be published.

Please complete the table below to indicate the contributions of all named authors to the figures.

Figure 1:

Yiqi Yang provided important comments on the figure and critically revised it.
Ziyang Lin substantial contributions to designing the figure and drawing the overall content.
Lin Quanyou is responsible for collecting and checking all references required for the figure.
Bei Weijian and Guo Jiao provided important revision suggestions and finally approved the version to be published.

Figure 2:

Yiqi Yang provided important comments on the figure and critically revised it.
Ziyang Lin substantial contributions to designing the figure and drawing the overall content.
Lin Quanyou is responsible for collecting and checking all references required for the figure.
Bei Weijian and Guo Jiao provided important revision suggestions and finally approved the version to be published.

Figure 3:

Figure 4:

Figure 5:

Figure 6:

Signed for and on behalf of the Author(s):	Print Name:	Date:
Yiqi Yang, Ziyang Lin, Quanyou Lin, Weijian Bei, Jiao Guo	Yiqi Yang, Ziyang Lin, Quanyou Lin, Weijian Bei, Jiao Guo	October 22, 2021
